# Hydrogen peroxide induces heme degradation and protein aggregation in human neuroglobin: roles of the disulfide bridge and hydrogen‐bonding in the distal heme cavity

**DOI:** 10.1111/febs.16581

**Published:** 2022-07-31

**Authors:** Giulia Di Rocco, Fabrizio Bernini, Gianantonio Battistuzzi, Antonio Ranieri, Carlo Augusto Bortolotti, Marco Borsari, Marco Sola

**Affiliations:** ^1^ Department of Life Sciences University of Modena and Reggio Emilia Italy; ^2^ Department of Chemical and Geological Sciences University of Modena and Reggio Emilia Italy

**Keywords:** aggregation, amyloid, electronic and MCD spectroscopies, fibril, hydrogen peroxide, neuroglobin

## Abstract

In the present study, human neuroglobin (hNgb) was found to undergo H_2_O_2_‐induced breakdown of the heme center at a much slower rate than other globins, namely in the timescale of hours against minutes. We investigated how the rate of the process is affected by the Cys46/Cys55 disulfide bond and the network of non‐covalent interactions in the distal heme side involving Tyr44, Lys67, the His64 heme iron axial ligand and the heme propionate‐7. The rate is increased by the Tyr44 to Ala and Phe mutations; however the rate is lowered by Lys67 to Ala swapping. The absence of the disulfide bridge slows down the reaction further. Therefore, the disulfide bond‐controlled accessibility of the heme site and the residues at position 44 and 67 affect the activation barrier of the reaction. Wild‐type and mutated species form β‐amyloid aggregates in the presence of H_2_O_2_ producing globular structures. Furthermore, the C46A/C55A, Y44A, Y44F and Y44F/C46A/C55A variants yield potentially harmful fibrils. Finally, the nucleation and growth kinetics for the aggregation of the amyloid structures can be successfully described by the Finke–Watzky model.

## Graphical abstract

Human neuroglobin (hNgb) undergoes H_2_O_2_‐induced breakdown of the heme center at a much slower rate than 5‐coordinated globins. The activation barrier of the reaction is affected by the disulfide bond‐controlled accessibility of the heme site and by the residues at positions 44 and 67. In the presence of H_2_O_2_, wild‐type and mutated hNgb form β‐amyloid aggregates producing globular structures. The C46A/C55A, Y44A, Y44F and Y44F/C46A/C55A variants yield potentially harmful fibrils.

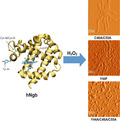

**Abbreviations**
AFM: atomic force microscopy
ATR‐FTIR: attenuated total reflectance‐FTIR
hNgb: human neuroglobin
ROS: reactive oxygen species
ThT: thioflavin T

## Introduction

**Fig. 1:**
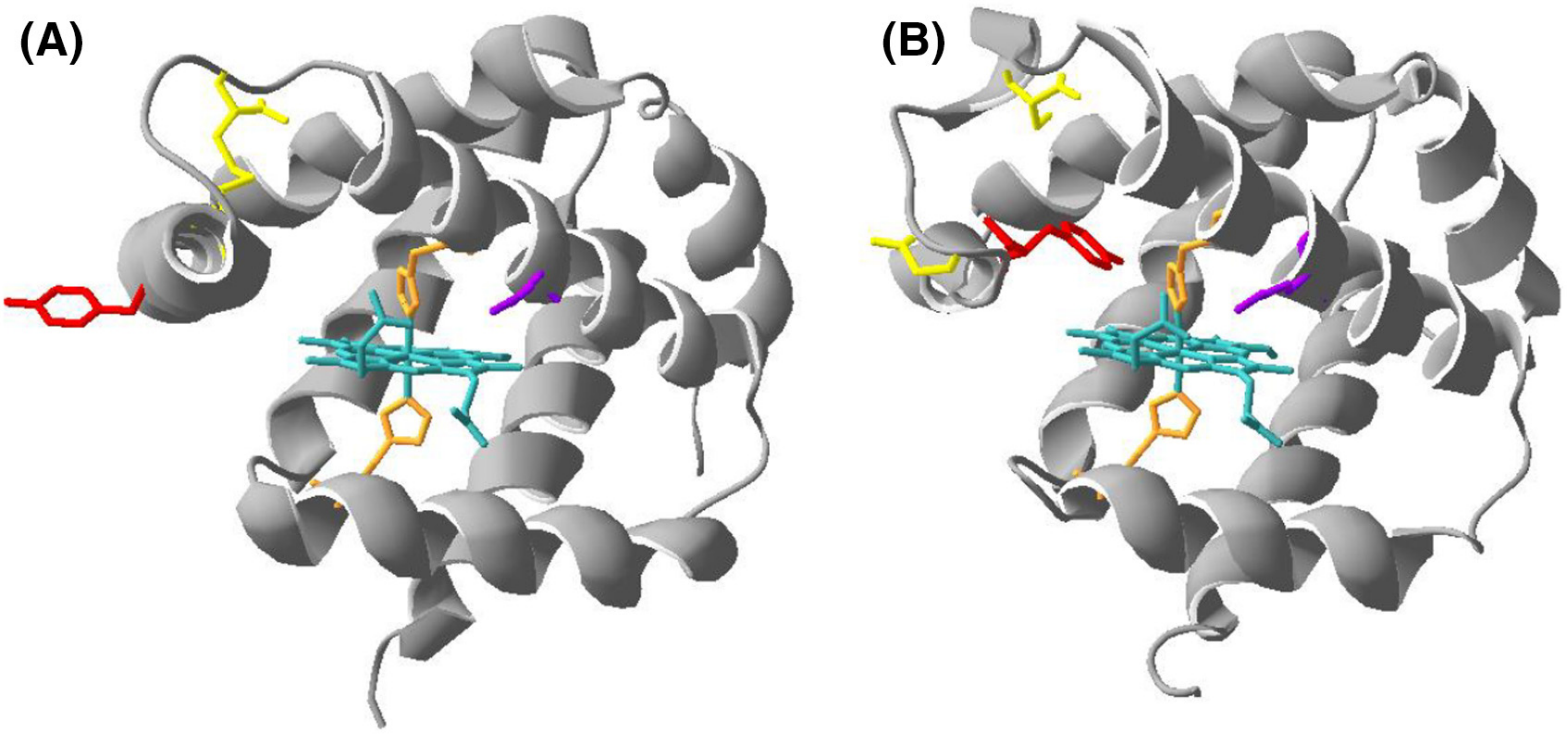
Cartoon representation of the structure of (A) wt hNgb (4mpm.pdb, chain A) featuring the disulfide bridge between Cys46 and Cys55 and of (B) the C46/C55S mutant of hNgb (1oje.pdb, chain B) without the disulfide bridge between Cys46 and Cys55. The heme group and the iron axial ligands His 64 and His 96 are represented in light blue and orange, respectively, whereas the residues that were mutated in the present study are represented in yellow (Cys 46 and Cys 55), red (Tyr 44) and violet (Lys 67).

In recent years, the neuroprotective role of human neuroglobin (hNgb) under conditions of oxidative stress has increasingly clearly been recognized [1, 2, 3, 4, 5, 6, 7, 8, 9]. hNgb is one of the most interesting members of the six‐coordinate His/His‐ligated heme *b*‐containing class of globins found in the last two decades in different organisms, including mammals, avians, fishes and amphibians [2, 3, 9]. hNgb is a 17‐kDa monomer featuring a typical globin fold [2, 10, 11, 12, 13] despite < 30% sequence identity with myoglobin (Fig. 1) [2, 12, 13, 14]. The six‐coordinate (His96/His64)‐axially ligated form of hNgb is in equilibrium with a minor five‐coordinate species in which His64 is detached from the iron atom. hNgb is abundant in the neurons of the hypothalamus and in the retinal cells (with concentrations up to 100 μm [15]), whereas it is present at a relatively low concentration (approximately 1 μm) in other parts of the central nervous system [16, 17, 18, 19, 20, 21, 22, 23, 24] and in non‐neuronal tissues such as the heart, gastrointestinal tract, haematopoietic stem cells, tumoral cells and endocrine tissues [2, 4, 18, 20, 25, 26, 27, 28]. The physiological role of hNgb remains an issue [2, 3, 9, 29]. The six‐coordinate heme iron and the high rate of autooxidation of the ferrous heme rule out its involvement in the storage/transport of O_2_ (and other ligands) typical of ordinary 5‐coordinate globins, although its function as O_2_ supplier to the nerve cells with respect to protecting them in case of hypoxia and ischemia has been proposed [2, 9, 15, 28, 29]. Strong evidence has been gained indicating that *in vivo* hNgb is overexpressed under conditions of oxidative stress, hypoxia and glucose deficiency, thereby suggesting a neuroprotective function, although this remains debated [1, 2, 3, 4, 6, 7, 8, 9, 29, 30]. In particular, hNgb overexpression would protect neurons from mitochondrial dysfunctions [2, 7, 8, 9, 31, 32] and neurodegenerative disorders, such as Alzheimer's disease [2, 9, 33, 34, 35, 36]. Other possible functions include scavenging of dangerous reactive nitrogen species and reactive oxygen species (ROS) and the conversion of harmful surplus of NO to ${\mathrm{NO}}_{3}^{-}$ [37, 38, 39, 40] and also the production of NO from ${\mathrm{NO}}_{2}^{-}$ for signalling events [39, 41, 42, 43, 44]. Moreover, hNgb reduces the ferric cytochrome *c* released in the cytosol, thus preventing apoptosis induced by the oxidative stress [2, 9, 24, 45, 46, 47, 48, 49, 50, 51]. Interestingly, because H_2_O_2_‐induced heme degradation in Ngb is considerably slower compared to other heme proteins, it has been converted into multifunctional enzymes that are capable of catalyzing dye‐decolorization and dehalogenation reactions using H_2_O_2_ as an oxidant [52, 53].

In the present study, we investigated the interaction of hNgb with hydrogen peroxide, the paradigmatic ROS [54], aiming to gain information on the response of this protein to an oxidizing agent that is easily formed under oxidative stress conditions. These findings may help our understanding of one important action underlying the protective role of this species against oxidative cell damage. We tackled this problem by investigating the native protein and a few mutants on key residues affecting the protein environment and the accessibility of the heme center to solvent. In particular, the double mutant C46A/C55A lacks the intramolecular disulfide bridge between these cysteine residues that forms under oxidizing conditions and is considered a determinant of the functional regulation of the protein [2, 55, 56, 57, 58, 59, 60, 61, 62, 63, 64, 65]. Removal of the disulfide bridge results in the strengthening of the axial heme iron bond with the distal His64 and the partial closure of the heme crevice. These changes decrease heme accessibility to solvent that in turn lowers the affinity of hNgb for exogenous ligands, such as O_2_, ${\mathrm{NO}}_{2}^{-}$ and CN^−^ [41, 42, 44, 55, 59, 60, 65, 66]. Conversely, the presence of the disulfide bridge significantly increases hNgb affinity for exogenous ligands as well as its activity as ${\mathrm{NO}}_{2}^{-}$ reductase [42]. Tyr44 and Lys67 are other key residues located in the heme distal side (Fig. 1). In the absence of the disulfide bridge, they take part in a hydrogen‐bonding and electrostatic network also involving the distal His64 and the heme propionate 7 [12, 59], which stabilizes the heme environment and limits the access of exogenous ligands to the heme iron providing an energy barrier for ligand binding prior to detachment of the distal His64 [67, 68, 69, 70]. Here, we replaced Tyr44 with an Ala and a Phe residue. Both variants lack the hydrogen‐bonding network yielded by Tyr44 in wild‐type (wt) hNgb, but differ in the steric hindrance of the substituting residue. The Y44A mutation would induce a larger accessibility of the distal heme side to solvent and exogenous ligands. Upon formation of the C46–C55 intramolecular disulfide bridge, Tyr44 moves out of the heme cavity [12, 59]. Therefore, we investigated the effect of these Tyr44 replacements on the interaction with H_2_O_2_ in the presence and absence of the abovementioned disulfide bridge by examining the single Y44A and Y44F variants and the triple Y44A/C46A/C55A and Y44F/C46A/C55A variants, respectively. Similarly, we examined the K67A and K67A/C46A/C55A variants. Moreover, we focused on the impact of these mutations on the H_2_O_2_‐induced protein aggregation and fibril formation. Ngb shows a certain ability to form amyloid fibrils spontaneously both in apo and holo form, even if with very slow kinetics [71]. The nucleation and growth kinetics of the protein aggregates were found to follow the Finke–Watzky model [72, 73]. Overall, these mutations demonstrate that the architecture of the distal site of hNgb controls the kinetics and thermodynamics of the hydrogen peroxide‐induced heme breakdown and protein aggregation.

## Results

### Effects of hydrogen peroxide on the electronic absorption spectra of wt and mutated hNgb

The absorption spectra of all species are almost identical and typical of a six‐coordinate ferric heme as indicated by the Soret band at 413 nm and the Q bands at 532 and 554(sh) nm (Fig. S1) [10, 38, 39]. In all cases, no five‐coordinate heme‐containing species were detected consistent with the low abundance (< 5%) of this form at neutral pH [74]. The Soret bands recorded over time after addition of 50 and 200 μm H_2_O_2_ are shown in Fig. S2. These H_2_O_2_ concentrations mimic a chronic/prolonged (timescale of days) and acute/transitory (timescale of minutes/hours) condition of extracellular oxidative stress, respectively [75]. The intensities of the Soret and the Q bands decrease to zero with time (Figs S1 and S2). No changes in wavelength and shape are observed, as well as no appearance of new signals. These results indicate that H_2_O_2_ causes progressive heme degradation. After about 30 h, the formation of a white milky precipitate is observed.

**Fig. 2:**
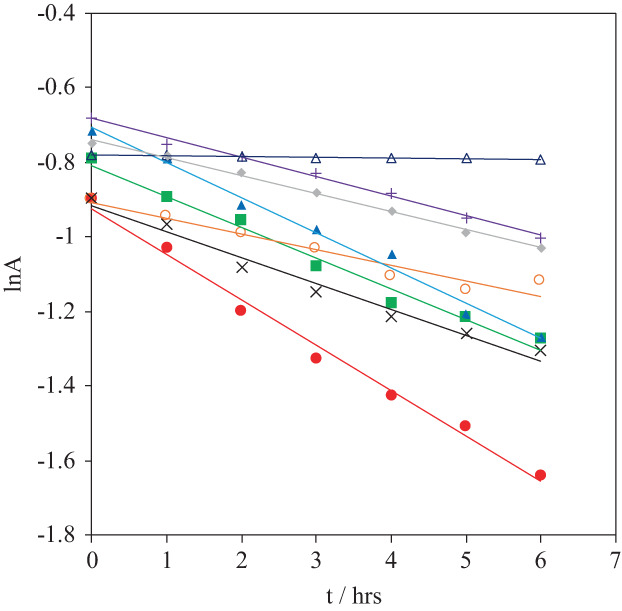
Plot of the absorbance of the Soret band at 413 nm vs. time in the presence of 200 μm H_2_O_2_: (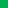) wt, (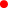) Y44A, (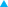) Y44F, (×) K67A, (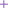) C46AC55A, (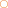) Y44A/C46A/C55A, (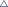) Y44F/C46A/C55A and (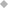) K67A/C46A/C55A. Protein concentration: 3–5 μm, 10 mm phosphate buffer plus 0.1 m NaCl (pH 7.4). *T* = 298 K. The same plots in the presence of 50 μm H_2_O_2_ are shown in Fig. S3.

The time course of the intensity decrease follows a pseudo first‐order kinetics (Fig. 2 and Fig. S3). The kinetic constants are listed in Table 1. Heme degradation is faster in 200 μm H_2_O_2_ compared to 50 μm H_2_O_2_, whereas, for a given H_2_O_2_ concentration, it slows down for the variants lacking the disulfide bridge (namely bearing the C46A/C55A mutations). Moreover, in these mutants, the Tyr44 to Ala and Phe and the Lys67 to Ala mutations exert a rate‐decreasing effect. By contrast, in the presence of the disulfide bridge, the reaction rate is increased by the Tyr44 to Ala and Phe mutations and decreased upon replacement of Lys67 with Ala.

**Table 1:** Pseudo first‐order kinetic constants for H_2_O_2_‐induced heme degradation for wt hNgb and its mutants in the presence of 50 and 200 μm H_2_O_2_. Values were obtained from the plots shown in Fig. 2 and Fig. S3. The *k* values are affected by an error of about ±0.005 h^−1^. Protein concentration: 3–5 μm, 10 mm phosphate buffer plus 0.1 m NaCl (pH 7.4). *T* = 298 K.

hNgb	*k*_50_/h^−1^	*k*_200_/h^−1^
wt	0.083	0.170
C46A/C55A	0.052	0.112
Y44A	0.122	0.236
Y44A/C46A/C55A	0.042	0.090
Y44F	0.094	0.189
Y44F/C46A/C55A	0.002	0.004
K67A	0.069	0.139
K67A/C46A/C55A	0.048	0.104

### Effects of hydrogen peroxide on the aggregation of wt and mutated hNgb Attenuated total reflectance‐FTIR (ATR‐FTIR) and thioflavin fluorescence spectra in the presence of H_2_O_2_

The ATR‐FTIR spectra of all species (Fig. S4) contain a single band for both Amide I and Amide II vibrational modes. The Amide I band (1600–1700 cm^−1^) originates for the most part from the stretching of the peptide C=O bonds of the protein backbone and is particularly sensitive to the amount of the various secondary structure motifs [76, 77, 78]. Amyloid fibrils show a characteristic band clustering between 1611 and 1630 cm^−1^, whereas, for native β‐sheet proteins, it is observed from 1630 to 1643 cm^−1^ [79]. For all species, H_2_O_2_ induces a broadening of the Amide I band that also becomes less symmetric, particularly in the lower wavenumber region, indicative of changes in the secondary structure. The second‐derivative spectra were calculated in the absence of hydrogen peroxide and after 24 h of incubation with 200 μm H_2_O_2_ (Fig. S5). Those obtained after incubation with H_2_O_2_ always show a significant increase in the signal in the region around 1620–1630 nm (not observed or negligible in the absence of H_2_O_2_), suggesting the formation of β‐sheet structures of amyloid type [76, 77, 78].

**Fig. 3:**
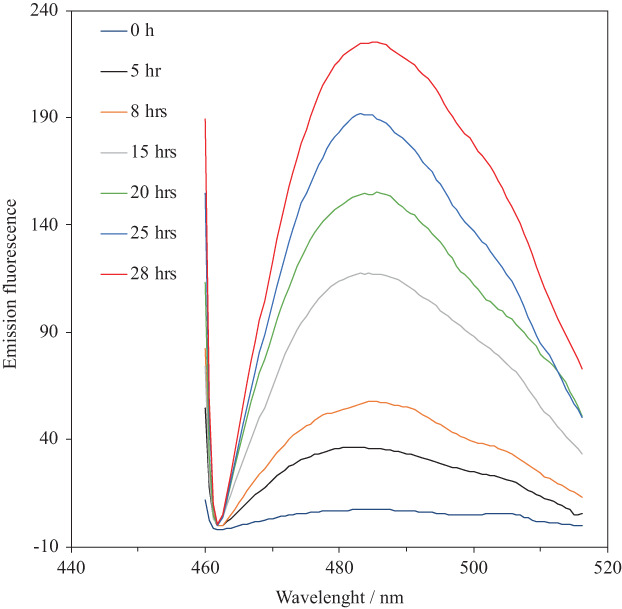
Time course of the thioflavin T (ThT) fluorescence spectrum in the presence of wt hNgb upon addition of 200 μm H_2_O_2_. The variants show a similar behaviour. Excitation at 450 nm, ThT concentration: 20 μm; protein concentration: 5 μm, 10 mm phosphate buffer plus 0.1 m NaCl (pH 7.4). *T* = 298 K.

**Fig. 4:**
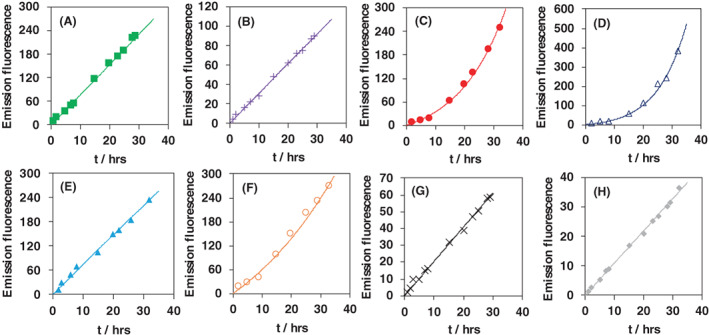
Plot of the ThT fluorescence emission intensity of the 485 nm band vs. time upon addition of 200 μm H_2_O_2_: (A) wt, (B) C46AC55A, (C) Y44A, (D) Y44AC46AC55A, (E) Y44F, (F)Y44FC46AC55A, (G) K67A and (H) K67AC46AC55A. ThT concentration: 20 μm; protein concentration: 5 μm, 10 mm phosphate buffer plus 0.1 m NaCl (pH 7.4). *T* = 298 K. Continuous lines are the best fit curves based on the Finke–Watzky model [72, 73].

Thioflavin T (ThT) is a widely exploited molecular probe used to investigate the mechanism of formation of β‐amyloid aggregates. ThT binding to β‐sheet‐containing structures, such as amyloid fibrils, is highly specific and induces a blue‐shift of the ThT fluorescence band maximum from 510 nm to 480–490 nm accompanied by an intensity increase [80, 81]. ThT added to hNgb and the present variants shows the fluorescence spectrum typical of the unbound form even after 24 h of incubation. By contrast, for all species, addition of H_2_O_2_ results in the appearance and progressive growth of the 485 nm band typical of the presence of amyloid aggregates (Fig. 3). The time course of such an intensity increase is shown in Fig. 4. Unfortunately, protein precipitation occurring after about 30 h incubation causes a fast decrease in the intensity of the fluorescence emission and prevents observation of the plateau phase corresponding to the end point for the conversion of the protein into amyloid aggregates.

The C46A/C55A, Y44A and Y44A/C46A/C55A variants feature a sort of induction time, followed by fast amyloid formation, whereas the wt, Y44F and Y44F/C46A/C55A variants show a more progressive fluorescence increase, and the K67A and K67A/C46A/C55A variants show almost no band formation. Invariably, the experimental data fit well with those calculated by applying the Finke–Watzky model (Fig. 4). Such a two‐step model for aggregate formation assumes a first nucleation step followed by an autocatalytic growth at the surface, namely [72, 73]:

$$\mathrm{nA}\overset{k_{1}}{\to }B_{n}\;n>2$$

$$B_{n}+A\overset{k_{2}}{\to }B_{n+1}$$

where *A* is the monomer and *B*_n_ is the oligomer formed by nucleation, which grows autocatalytically. A number of protein aggregation events leading to neurological diseases involving amyloid (Alzheimer's disease), α‐synuclein (Parkinson's disease) and polyglutamin (Huntington's disease) follow the kinetic law obtained from this model (Eqns (1), (2), (3)) [73]:

(1) $$-d\left[A\right]/\mathrm{dt}=k_{1}\left[A\right]+k_{2}\left[A\right]\left[B_{y}\right]\;B_{y}=\sum_{n}B_{n},$$

(2) $${\left[A\right]}_{t}=\left[\left(k_{1}/k_{2}\right)+{\left[A\right]}_{0}\right]/\left[1+\left(k_{1}/k_{2}{\left[A\right]}_{0}\right)\;\exp\left[\left(k_{1}+k_{2}{\left[A\right]}_{0}\right)t\right]\right]$$

(3) $${\left[B\right]}_{t}={\left[A\right]}_{0}-\left[\left(k_{1}/k_{2}\right)+{\left[A\right]}_{0}\right]/\left[1+\left(k_{1}/k_{2}{\left[A\right]}_{0}\right)\exp\left[\left(k_{1}+k_{2}{\left[A\right]}_{0}\right)t\right]\right]$$

The *k*_1_ and *k*_2_ values obtained from the data fit are listed in Table 2.

**Table 2:** Kinetic constants for H_2_O_2_‐induced nucleation (*k*_1_) and autocatalytic growth at a surface (*k*_2_) for wt hNgb and its mutants according to the Finke–Watzky model [72, 73]. Values were obtained from the plots shown in Fig. 4. The *k* values are affected by a relative error of about ±8%. Protein concentration: 3 μm, 10 mm phosphate buffer plus 0.1 m NaCl (pH 7.4). *T* = 298 K. For sake of comparison, the kinetic data for aggregation of Aβ_42_, α‐synuclein and polyglutamin are reported from Morris *et al*. [73].

hNgb	*k*_1_/h^−1^	*k*_2_/mm ^−1^·h^−1^
wt	1.52 × 10^−3^	0.51
C46A/C55A	0.62 × 10^−3^	0.51 × 10^−2^
Y44A	0.49 × 10^−3^	13.1
Y44A/C46A/C55A	0.29 × 10^−3^	21.7
Y44F	1.50 × 10^−3^	0.11 × 10^−2^
Y44F/C46A/C55A	1.05 × 10^−3^	5.61
K67A	0.41 × 10^−3^	0.72 × 10^−2^
K67A/C46A/C55A	0.22 × 10^−3^	0.37 × 10^−2^
αβ_42_	1.0 × 10^−4^	5.7 × 10^−2^
αβ_42_ + Zn(II)	8.3 × 10^−4^	6.3 × 10^−2^
α‐Synuclein	0.48 × 10^−4^	40
Polyglutammine	2.6 × 10^−3^	2.3 × 10^−2^

### Atomic force microscopy (AFM) study of protein aggregates

**Fig. 5:**
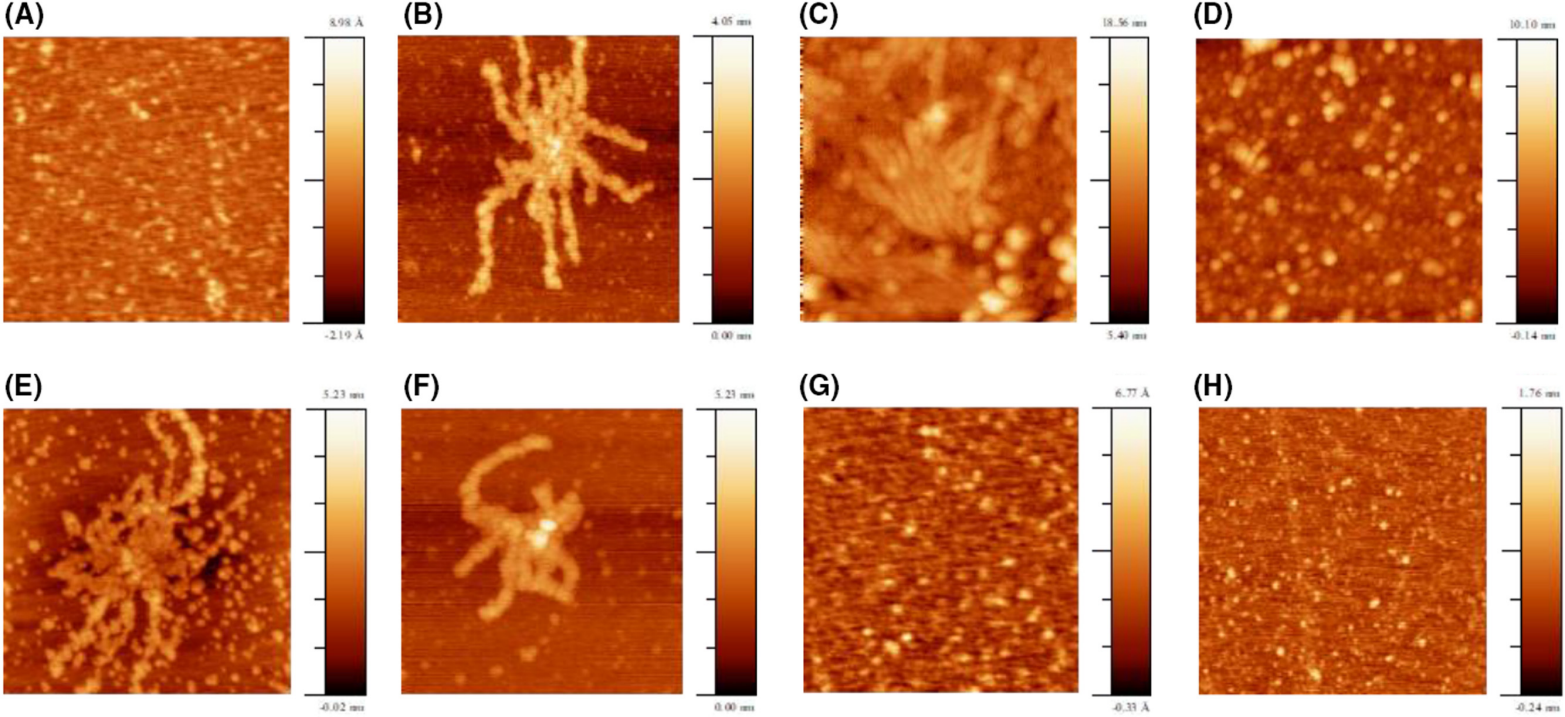
Topographic AFM images of protein aggregates formed by hNgb and variants subjected to incubation with H_2_O_2_: (A) wt, (B) C46AC55A, (C) Y44A, (D) Y44AC46AC55A, (E) Y44F, (F) Y44FC46AC55A, (G) K67A and (H) K67AC46AC55A. Protein samples (5 μm in 10 mm phosphate buffer plus 0.1 m NaCl, pH 7.4, and 200 μm H_2_O_2_) were aged at 20 °C in the dark for 24 h.

In the absence of H_2_O_2_, no significant aggregates on mica surface are detected for all species within 24 h. By contrast, protein aggregates form after 24 h of incubation with 200 μm H_2_O_2_. The AFM images are shown in Fig. 5 and Fig. S6. wt hNgb yields several small globular aggregates, often poorly defined, but no fibrils. Their largest height is 0.45 nm, although the large majority of the aggregates do not exceed 0.2 nm (Fig. S7). The C46A/C55A variant lacking the disulfide bridge also shows numerous small aggregates, most of which are about 1.1–1.2 nm (maximum of 2 nm) in height; however, a few well developed fibrils are observed with a height between 2.0 and 2.1 nm and lengths from 200 to 300 nm (Fig. S7). The Y44A variant, instead, forms well defined and large globular aggregates (2.5–3.5 nm, maximum of 8 nm in height), but also agglomerate structures and squat fibrillary bodies, mostly aggregated, the latter with heights between 2.5 and 3.5 nm and lengths from 50 to 100 nm (Fig. S7). The variant containing the same mutation but deprived of the disulfide bridge, namely Y44A/C46A/C55A, yields large and well defined globular aggregates as described above (average 4 nm, maximum of 8 nm in height), some rare agglomerate structures but no fibrils at all (Fig. S7). Insertion of a Phe in place of Tyr44 in the Y44F variant yields longer fibrils compared to Y44A. In particular, the globular aggregates are numerous and smaller than above (average height of 2.0–2.5 nm), although well developed and often overlapped fibrillary structures are observed, 2.0–2.5 nm high and lengths on average > 200 nm that can reach up to 500 nm (Fig. S7). Upon cleavage of the disulfide bond in the Y44F/C46A/C55A variant only minor changes are observed. In particular, the globular aggregates are lower (average height 1.0–1.2 nm) such as the fibrillary structures (1.2–1.6 nm), which show lengths from 300 to slightly above 500 nm (Fig. S7). Similar to wt hNgb, both K67A and K67A/C46A/C55A yield small globular aggregates (with an average height of 0.3–0.4 nm and 1–1.2 nm for K67A and K67A/C46A/C55A, respectively), but no fibrils (Fig. S7).

## Discussion

### Kinetics and mechanism of H_2_O_2_ ‐induced heme degradation in hNgb

The mechanism of H_2_O_2_‐induced oxidative degradation of the heme center in globins starts with the formation of a Fe(III)‐hydroperoxide intermediate that yields the α‐meso‐hydroxy‐heme group, which then evolves in verdoheme (upon CO release) and afterwards is further oxidized to biliverdin [82, 83, 84, 85, 86, 87, 88]. One of the physiological roles hypothesized for hNgb is the protection of the neuron and retinal cell from oxidative stress with an as yet unknown mechanism [1, 2, 3, 4, 6, 7, 8, 29, 30, 38, 89] that, as for the other globins [90, 91, 92, 93], could involve heme degradation. The generally accepted mechanism by which pentacoordinate globins, such as myoglobin and haemoglobin, react with hydrogen peroxide [82, 83] involves the oxidation of the ferrous or ferric heme of the globin by hydrogen peroxide (or peroxides) to a ferryl species. The latter, unlike the true peroxidase enzymes, is very unstable and, in the absence of substrate, leads to oxidative modifications or degradation of the heme itself or the protein. The proposed protective role of hNgb, however, would conflict with its documented resistance to the oxidative stress attributed to the unfavored formation of the ferryl heme center as a result of the presence of the two axial His ligands to the heme iron [38, 94]. However, we note that the above finding is based on experiments in which relatively brief reaction times of hNgb with H_2_O_2_ were allowed (< 1 h) [38, 94]. This timescale was chosen by analogy to myoglobin and haemoglobin that react completely with H_2_O_2_ within minutes [83, 95, 96, 97, 98, 99]. In hNgb, the six‐coordinate heme center and the steric hindrance of the polypeptide matrix surrounding the heme make the heme center scarcely accessible and reactive to exogenous ligands. Here, we found that these features do not prevent heme degradation and subsequent iron ion release by H_2_O_2_ but make it dramatically much slower compared with the other globins. Moreover, the reaction rate is affected by mutation of both Tyr44 and Lys67. Indeed, the pseudo first‐order kinetic constants obtained from the time course of the H_2_O_2_‐induced bleaching of the Soret band (Fig. 2 and Table 1) decrease in the order: Y44A > Y44F > wt > K67A > C46A/C55A > K67A/C46A/C55A > Y44A/C46A/C55A> > Y44F/C46A/C55A for both H_2_O_2_ concentrations investigated. The last mutant is particularly resistant to the oxidative action of H_2_O_2_, showing a very limited heme degradation even after 24 h. These mutational effects on the kinetic constants could be related to the accessibility of the heme center to solvent, for which the decrease is expected to slow down heme degradation. Indeed, heme accessibility is reduced by the removal of the disulfide bridge as it closes the heme crevice, and it is also affected by the steric hindrance and the arrangement of the Tyr44 and Lys67 side chains. A rough correlation can be established between the kinetic constants and the volume of the heme crevice (Fig. S8). Y44F/C46A/C55A is a clear outlier and the other data are rather scattered, suggesting that other factors also control the rate of the oxidative heme degradation. Indeed, the kinetic constants must also conceivably be related to the amount of five‐coordinate heme center, which is the only species able to interact with H_2_O_2_ to yield the hydroperoxo derivative and eventually the ferryl center needed for heme degradation. Such a content is known to decrease with removal of the disulfide bridge and may also be subjected to mutational effects [12, 57, 62]. In addition, other factors, such as the availability of residues able to form hydrogen bonds and the presence of hydrophobic regions in the heme pocket, could play a relevant role.

We note that the kinetic constants for 200 μm H_2_O_2_ are approximately twice those for 50 μm H_2_O_2_. Therefore, despite only two data points being available, it is unlikely that the reaction is of the first order with respect to H_2_O_2_. Therefore, the mechanism of heme oxidation by H_2_O_2_ is complex. Here, neither hydroperoxide nor verdoheme intermediates (showing absorptions at about 700 nm) typical of heme breakdown for the other globins and heme proteins [100, 101] have been observed in the UV‐visible spectra of hNgb incubated with H_2_O_2_. However, these bands could be low‐intensity because of the kinetic lability of these species and the slowness of the first oxidation step, which does not allow the accumulation of the reaction intermediates. Accordingly, we note that a broad feature at about 700 nm appears with time in the second derivative spectra of hNgb in the presence of H_2_O_2_ (Fig. S9). Therefore, the presence of the verdoheme intermediate in the heme breakdown of hNgb cannot be excluded [96]. Its concentration during the reaction could be small as a result of its fast transition to biliverdin. These observations suggest that the heme group of hNgb undergoes H_2_O_2_‐induced oxidative degradation, possibly sharing the mechanism of the other globins, although it features a larger resistance that is kinetic in nature because the process is much slower.

### Aggregation of hNgb upon H_2_O_2_ ‐induced heme breakdown

Ngb is capable of spontaneously forming amyloid fibrils with very slow kinetics [71]. In the presence of H_2_O_2_, however, the rate of this process increases considerably and is a function of residue replacement around the heme center. For all of the investigated species, the β‐sheet content is negligible or very low, as can be seen from the second‐derivative AT‐FTIR spectra (Fig. S5). Such content increases upon H_2_O_2_ addition and the consequent protein aggregation. The ThT fluorescence spectra indicate the formation of amyloid type structures for which aggregation kinetics differ for the various species (Figs 3 and 4 and Table 2). The *k*_1_ values are rather similar, whereas the *k*_2_ values are affected by some mutation‐induced differences. The presence of the disulfide bridge does not affect the kinetics of amyloid formation (Table 2). Moreover, the *k*_1_ and *k*_2_ values do not correlate with the ability of the protein to yield fibrils: indeed, the species that do not produce fibrillar structures (wt, Y44A/C46A/C55A, K67A and K67A/C46A/C55A) show kinetic constants that are scattered within the protein series.

AFM measurements (Fig. 5 and Fig. S6) show that fibrils are formed by Y44F, Y44F/C46A/C55A, Y44A and C46A/C55A incubated with 200 μm H_2_O_2_. The disulfide bridge does not exert a univocal effect on fibril formation and there is no relationship between the yield of the aggregates and their morphology. wt hNgb produces amyloid aggregates with a high yield but these do not organize to yield fibrils. They do so only in the absence of the disulfide bridge (the C46/C55 variant), namely under reducing instead of oxidizing conditions. Consistently, the formation or breaking of a disulfide bridge has often been closely associated with amyloid formation for several proteins. [102, 103, 104, 105]. Interestingly, the Y44F/C46A/C55A, although showing very slow oxidation kinetics, yields similar amount of fibrillary structures within the same time scale compared to wt Ngb and the other mutants. This can tentatively be related to the formation of mixed fibrillary structures involving both degraded and intact species, with the former acting as sort of aggregation promoter [106, 107, 108].

Fibrils are responsible of plaque formation in Alzheimer's disease and other neurodegenerative diseases. However, we note that the mutants studied here, although presently not included among those possessing a physio‐pathological relevance [2], do yield fibrils if subjected to oxidative stress. Therefore, ROS could in principle poise other as yet unknown hNgb mutants for β‐amyloid fibril formation.

## Conclusions

The present study provides compelling evidence indicating that hNgb is more resistant to the oxidative action of H_2_O_2_ compared to the other globins (i.e. haemoglobin and myoglobin), but is nonetheless subjected to heme breakdown, although at a much slower rate. Such behaviour is related mainly to the presence of a heme center in equilibrium between a largely prevailing six‐coordinated state and a five‐coordinated one. The reaction with H_2_O_2_ is slower for the former state compared to the latter because of the need for ligand substitution. The absence of the disulfide bond in the variant containing the C44A and C55A mutations results in a further lowering of the reaction rate because of the conformational rearrangement that decreases the solvent accessibility of the heme center. wt Ngb and all of the variants investigated here yield β‐amyloid aggregates in the presence of H_2_O_2_. However, only the aggregates formed by the Y44F and Y44A variants evolve towards the production of fibrils, whereas the wt variant only does so upon cleavage of the disulfide bridge. It therefore appears that hNgb behaves like a two‐faced Janus. On the one hand, under conditions of chronic oxidative disorders, it may undergo heme degradation, thereby consuming ROS and protecting the cell from further damage. On the other, if the disulfide bridge breaks or some mutated form are present, it may aggregate to form β‐amyloid fibrils that produce plaques, thereby contributing to the onset of Alzheimer's disease or retinal amyloidosis.

## Materials and methods

### Materials

All chemicals were reagent grade. Tris(hydroxymethyl)aminomethane (Tris) was purchased from Sigma‐Aldrich (St Louis, MO, USA). Sodium mono‐hydrogen phosphate, sodium di‐hydrogen phosphate, sodium chloride and sodium perchlorate were purchased from Carlo Erba Reagenti (Milan, Italy). Water was purified through a Milli‐Q Plus Ultrapure Water System coupled with an Elix‐5 Kit (Millipore, Burlington, MA, USA). The water resistivity was > 18 MΩ cm.

### Protein expression and isolation

hNgb (GeneBank reference sequence AF422796) was expressed in *Escherichia coli* as reported previously [56]. The synthetic genes for all the Ngb variants (Y44A, Y44F, K67A, C46A/C55A, Y44A/C46A/C55A, Y44F/C46A/C55A and K67A/C46A/C55A) were purchased from Integrated DNA Technologies (Coralville, IA, USA) and subcloned in the prokaryotic expression vector pLATE11 (aLICator Ligation Independent Cloning and Expression System; Thermo Scientific, Waltham, MA, USA). The amplification by PCR produced the correct expected band for each clone. The recombinant clones were checked by PCR colony screening and sequenced for a further confirmation. The positive clones were then transformed into competent BL21 (DE3) *E. coli* strain containing the T7RNA polymerase gene under the control of the isopropyl thio‐β‐d‐galactoside‐inducible lacUV5 promoter. Protein expression was carried on at 25 °C in LB medium containing 100 μg·mL^−1^ ampicillin and 5 μg·mL^−1^ hemin solution for all the species. Expression was induced by 0.5 mm isopropyl thio‐β‐d‐galactoside. After 14–18 h, the reddish bacteria were collected by centrifugation and resuspended in 25 mm Tris (pH 8) and 10 mm EDTA. Cells were disrupted by sonication using a Microson ultrasonic cell disruptor (Misonix, Inc., Farmingdale, NY, USA) performing 10 cycles of 1 min each maintaining the cells in ice to avoid protein denaturation. Cell debris was removed by centrifugation for 10 min at 15000 ***g*** at 4 °C collecting the supernatant that contained the protein solution. hNgb was then precipitated in the presence of 4 m (NH_4_)_2_SO_4_ and, after a proper salting out procedure by two dialysis changes, purified by anionic‐exchange chromatography (DE‐52; Whatman, Little Chalfont, UK) followed by a size‐exclusion chromatography (Superdex G‐75; GE Healthcare Life Sciences, Piscataway, NJ, USA) using an AKTA prime GE Healthcare system [56]. Approximately 8 mg of pure hNgb were obtained from 1 L of culture. The purity and the concentration of the proteins was tested by SDS gel electrophoresis (15% acrylamide), which yielded a single band in the gel at around 17 kDa for all of the sample testeds corresponding to the calculated neuroglobin masses [2, 10, 23].

### Spectroscopic measurements

Electronic absorption spectra were recorded with a model V‐570 spectrophotometer (Jasco, Tokyo, Japan). All experiments were carried out at 25 °C with 3–5 μm protein solutions freshly prepared before use in 10 mm phosphate buffer plus 0.1 m NaCl (pH 7.4). The protein concentration was checked spectrophotometrically using ε_412_ = 129 000 m ^−1^·cm^−1^ for all the proteins [44]. Hydrogen peroxide was added to the above solution to a final concentration of 50 and 200 μm. The experiments were repeated at least five times and the average kinetic constants for H_2_O_2_‐induced heme degradation were affected by an uncertainty of ±0.005 h^−1^ (maximum deviation).

ThT fluorescence spectra for wt and mutated hNgb were obtained with a model FP‐6000 spectrofluorimeter (Jasco). The spectra were recorded between 460 and 600 nm with excitation at 450 nm. Protein solutions, typically 3–5 μm, were made in 10 mm phosphate buffer plus 0.1 m NaCl (pH 7.4). Hydrogen peroxide was added to the above solution to a final concentration of 200 μm. ThT was added to the protein solution 10 min prior to fluorescence measurements aiming to minimize the possible interaction with H_2_O_2_. However, no fluorescence emission signal is observed in the range 480–490 nm (excitation at 450 nm) when ThT is mixed with H_2_O_2_. Also in this case, the experiments were performed at least five times and kinetic constants for H_2_O_2_‐induced nucleation (*k*_1_) and autocatalytic growth at a surface (*k*_2_) were calculated by fitting the Finke–Watzky model [72, 73] and comprise average values, with the associated errors being the maximum deviations as above.

ATR‐FTIR spectra were collected on a model FT‐IR 4700 spectrophotometer (Jasco) equipped with the ATR reflection device ATR Pro One (Jasco). The samples were obtained by dialysis to remove the buffer and NaCl and treated with 200 μm H_2_O_2_ for 24 h. Ten microlitres of the solution was then deposited on ATR device and dried before the measurement.

AFM measurements were carried out in the tapping mode, on the same solutions used for the fluorescence spectra, which were deposited on a mica foil and air‐dried at room temperature for 10–15 min. Protein samples (5 μm in 10 mm phosphate buffer plus 0.1 m NaCl, pH 7.4, and eventually 200 μm H_2_O_2_) were aged at 20 °C in the dark for 24 h. Approximately 20 μL of diluted sample (1 : 100) was deposited onto the freshly cleaved Mg^2+^ treated mica surfaces at room temperature (approximately 25 °C). The sample was incubated for 10 min in a water vapour saturated environment, then rinsed with ultrapure water and finally dried and stored in a flow of ultrapure dry nitrogen for at least 30 min at room temperature to remove the presence of water molecules at the surface.

The AFM analysis was performed by using a Multimode Nanoscope III atomic force microscope (Digital Instruments, Santa Barbara, CA, USA) with a Nanonis AFM control system (Nanonis – SPECS Zurich GmbH, Zurich, Switzerland) equipped with two oscillation controller modules (with digitally integrated phase‐locked loop/lock‐in). A nPoint closed‐loop multimode scanner (nPoint, Inc., Madison, WI, USA) with a C‐300 DSP closed‐loop controller was used as fine actuator of the tip–sample relative motion. Bruker (Camarillo, CA, USA) RTESPA‐150 and Nanoworld (Neuchâtel, Switzerland) Arrow‐EFM type rectangular probes working in intermittent contact mode were used for the morphological characterization.

### Computational analysis

The structures of the Ngb mutants were calculated by homology modelling using the swiss‐model workspace [109], with the crystal structure of human neuroglobin (4mpm.pdb, chain A [12]) serving as a template, and used for calculations for the wt species. The volume of the heme cavity for each Ngb species was estimated using castp [110]. Note that the cavity volume calculation neglects the heme center.

## Conflict of interest

The authors declare no conflict of interest.

## Author contributions

GDR and FB performed the experiments. GDR, FB, GB, AR and MB analyzed data. GB, AR and MB planned the experiments. GB, AR, CAR, MB and MS wrote the paper.

## Supporting information


**Fig. S1.** UV‐visible spectra for wt hNgb without H_2_O_2_ (black line) and after 22 h of incubation with 200 μm H_2_O_2_ (red line).
**Fig. S2.** Soret bands for spectra of hNgb and variants recorded over time after addition of H_2_O_2_ 50 μm (A to D) and 200 μm (E to H).
**Fig. S3.** Plot of the absorbance of the Soret band at 413 nm vs. time in the presence of 50 μm H_2_O_2_ (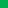) wt, (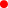) Y44A, (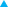) Y44F, (×) K67A, (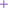) C46AC55A, (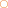) Y44A/C46A/C55A, (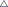) Y44F/C46A/C55A and (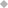) K67A/C46A/C55A.
**Fig. S4.** ATR‐FTIR spectra showing the Amide I and Amide II bands recorded over time after addition of H_2_O_2_ 200 μm for (A) hNgb wt, (B) Y44A, (C) Y44F and (D) K67A mutant with (black lines) and without (C46AC55A mutations, red lines) disulfide bridge.
**Fig. S5.** Second‐derivative ATR‐FTIR spectra of the Amide I band recorded over time after addition of H_2_O_2_ 200 μm for (A) hNgb wt, (B) Y44A, (C) Y44F and (D) K67A mutant with (black lines) and without (C46AC55A mutations, red lines) disulfide bridge.
**Fig. S6.** Morphological AFM images of protein aggregates formed by hNgb and variants subjected to incubation with H_2_O_2_: (A) wt, (B) C46AC55A, (C) Y44A, (D) Y44AC46AC55A, (E) Y44F, (F) Y44FC46AC55A, (G) K67A and (H) K67AC46AC55A.
**Fig. S7.** Topographic AFM images of protein aggregates formed by hNgb (a) wt, (b) C46AC55A, (c) Y44A, (d) Y44AC46AC55A, (e) Y44F, (f) Y44AC46AC55A, (g) K67A and (h) K67AC46AC55A subjected to incubation with H_2_O_2_ and section height of selected aggregates.
**Fig. S8.** Plot of *k*_200_ vs. the volume of the crevice where heme is placed (*V*_hc_).
**Fig. S9.** Seecond derivative electronic absorption spectra of wt hNgb interacting with H_2_O_2_ at *t* = 0 (black) and after 6 h of incubation (red).

## Data Availability

The data presented in this study are available on request from the corresponding authors.
