# Supplementary material for: Hydrogen peroxide induces heme degradation and protein aggregation in human neuroglobin: roles of the disulfide bridge and hydrogen‐bonding in the distal heme cavity
Source: FEBS J. 2022 Jul 31;290(1):148–61. doi: 10.1111/febs.16581 (PMC10087938; doi:10.1111/febs.16581)
Supplement: Supplementary file 1 — Fig. S1. UV‐visible spectra for wt hNgb without H2O2 (black line) and after 22 h of incubation with 200 μm H2O2 (red line). Fig. S2. Soret bands for spectra of hNgb and variants recorded over time after addition of H2O2 50 μm (A to D) and 200 μm (E to H). Fig. S3. Plot of the absorbance of the Soret band at 413 nm vs. time in the presence of 50 μm H2O2 () wt, () Y44A, () Y44F, (×) K67A, () C46AC55A, () Y44A/C46A/C55A, () Y44F/C46A/C55A and () K67A/C46A/C55A. Fig. S4. ATR‐FTIR spectra showing the Amide I and Amide II bands recorded over time after addition of H2O2 200 μm for (A) hNgb wt, (B) Y44A, (C) Y44F and (D) K67A mutant with (black lines) and without (C46AC55A mutations, red lines) disulfide bridge. Fig. S5. Second‐derivative ATR‐FTIR spectra of the Amide I band recorded over time after addition of H2O2 200 μm for (A) hNgb wt, (B) Y44A, (C) Y44F and (D) K67A mutant with (black lines) and without (C46AC55A mutations, red lines) disulfide bridge. Fig. S6. Morphological AFM images of protein aggregates formed by hNgb and variants subjected to incubation with H2O2: (A) wt, (B) C46AC55A, (C) Y44A, (D) Y44AC46AC55A, (E) Y44F, (F) Y44FC46AC55A, (G) K67A and (H) K67AC46AC55A. Fig. S7. Topographic AFM images of protein aggregates formed by hNgb (a) wt, (b) C46AC55A, (c) Y44A, (d) Y44AC46AC55A, (e) Y44F, (f) Y44AC46AC55A, (g) K67A and (h) K67AC46AC55A subjected to incubation with H2O2 and section height of selected aggregates. Fig. S8. Plot of k 200 vs. the volume of the crevice where heme is placed (V hc). Fig. S9. Seecond derivative electronic absorption spectra of wt hNgb interacting with H2O2 at t = 0 (black) and after 6 h of incubation (red). [file FEBS-290-148-s001.pdf]

SUPPORTING INFORMATION

Hydrogen Peroxide Induces Heme Degradation and Protein Aggregation in Human Neuroglobin: Roles of the Disulfide Bridge and the H-bonding in the Distal Heme Cavity

Giulia Di Rocco[1], Fabrizio Bernini[2], Gianantonio Battistuzzi[2,*], Antonio Ranieri[1,*], Carlo Augusto Bortolotti[1], Marco Borsari[2] and Marco Sola[2]

[1] *Department of Life Sciences, University of Modena and Reggio Emilia, via Campi 103, 41125 Modena, Italy*

[2] *Department of Chemical and Geological Sciences, University of Modena and Reggio Emilia, via Campi 103, 41125 Modena, Italy*

*****corresponding authors. e-mail: gianantonio.battistuzzi@unimore.it, antonio.ranieri@unimore.it

Running title: H_2O_2–induced aggregation and heme degradation in neuroglobin

SUPPORTING INFORMATION

Fig. SI 1. UV-Vis spectra for wt hNgb without H_2O_2 (black line) and after 22-hours incubation with 200 μM H_2O_2 (red line). Protein concentration: 3.5 μM, 10 mM phosphate buffer plus 0.1 M NaCl, pH 7.4. T = 298 K.

Fig. SI 2: Soret bands for spectra of hNgb and variants recorded over time after addition of H_2O_2 50 μM (A to D) and 200 μM (E to H). Continuous and dashed lines are referred to 0 hrs and 6 hrs after the injection of H_2O_2, respectively. Spectra A and E are for hNgb wt, B and F for the Y44A mutant, C and G for the Y44F mutant, and D and H for the K67A mutant with (black lines) and without (C46AC55A mutations, red lines) disulfide bridge.

Fig. SI 3: Plot of the absorbance of the Soret band at 413 nm vs. time in the presence of 50 μM H_2O_2 (<) wt, (●) Y44A, (▲) Y44F, (×) K67A, (+) C46AC55A, (○) Y44A/C46A/C55A, (△) Y44F/C46A/C55A, (◆) K67A/C46A/C55A. Protein concentration: 3-5 μM, 10 mM phosphate buffer plus 0.1 M NaCl, pH 7.4. T = 298 K.

Fig. SI 4: ATR-FTIR spectra showing the Amide I and Amide II band recorded over time after addition of H_2O_2 200 μM for (A) hNgb wt, (B) Y44A, (C) Y44F, and (D) K67A mutant with (black lines) and without (C46AC55A mutations, red lines) disulfide bridge. Continuous and dashed lines are referred to 0 hrs and 24 hrs after injection of H_2O_2, respectively. Protein solutions (concentration 3 μM, pH 7.4) were subjected to Ar drying before measurement, T = 298 K.

Fig. SI 5: 2nd-derivative ATR-FTIR spectra of the Amide I band recorded over time after addition of H_2O_2 200 μM for (A) hNgb wt, (B) Y44A, (C) Y44F, and (D) K67A mutant with (black lines) and without (C46AC55A mutations, red lines) disulfide bridge. Continuous and dashed lines are referred to 0 hrs and 24 hrs after injection of H_2O_2, respectively. Protein solutions (concentration 3 μM, pH 7.4) were subjected to Ar drying before measurement, T = 298 K.

Fig. SI 6: Morphological AFM images of protein aggregates formed by hNgb and variants subjected to incubation with H_2O_2: (A) wt, (B) C46AC55A, (C) Y44A, (D) Y44AC46AC55A, (E) Y44F, (F)

Y44FC46AC55A, (G) K67A, (H) K67AC46AC55A. Protein samples (5 μM in 10 mM phosphate buffer plus 0.1 M NaCl at pH 7.4 and 200 μM H_2O_2) were aged at 20 °C in the dark for 24h.

Fig. SI 7: Topographic AFM images of protein aggregates formed by hNgb (a) wt, (b) C46AC55A, (c) Y44A, (d) Y44AC46AC55A, (e) Y44F, (f) Y44AC46AC55A, (g) K67A, (h) K67AC46AC55A subjected to incubation with H_2O_2 and section height of selected aggregates. The protein samples (5 μM in 10 mM phosphate buffer plus 0.1 M NaCl at pH 7.4 and 200 μM H_2O_2) were aged at 20 °C in the dark for 24h.

Fig. SI 8. Plot of k_{200} vs. the volume of the crevice where heme is placed (V_{hc}). V_{hc} is calculated starting from the PDB structure of hNgb wt. The cavity volume calculation does not take the heme into account.

Fig. SI 9. 2nd derivative electronic absorption spectra of wt hNgb interacting with H_2O_2 at t = 0 (black) and after 6 hour incubation (red). Protein concentration: 3 μM, 10 mM phosphate buffer plus 0.1 M NaCl, pH 7.4. T = 298 K.

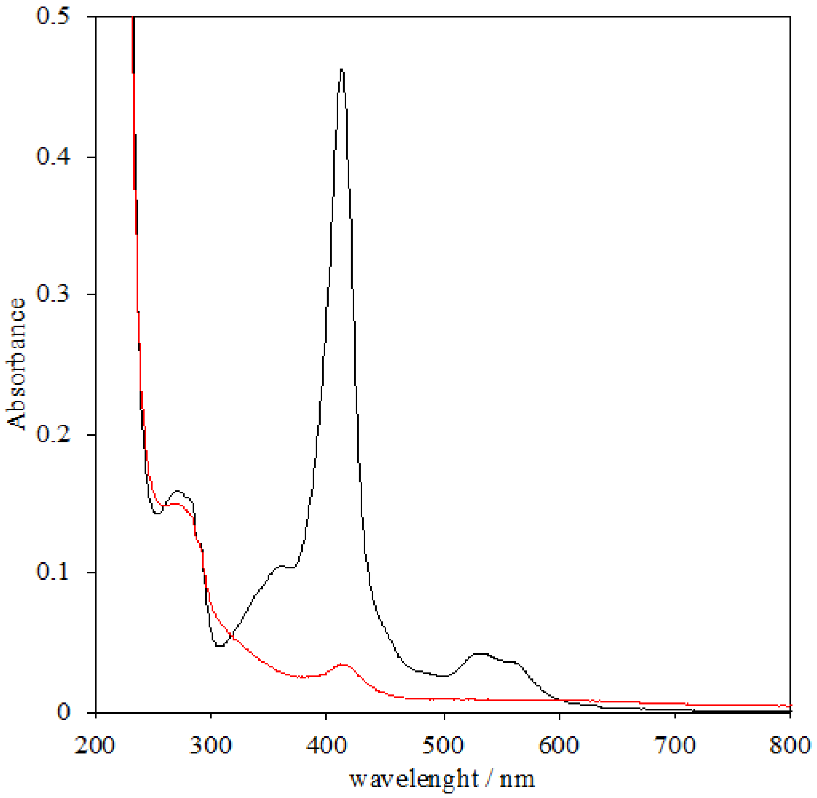

Fig. SI 1: UV-Vis spectra for wt hNgb without H_2O_2 (black line) and after 22-hours incubation with 200 µM H_2O_2 (red line). Protein concentration: 3.5 µM, 10 mM phosphate buffer plus 0.1 M NaCl, pH 7.4. T = 298 K.

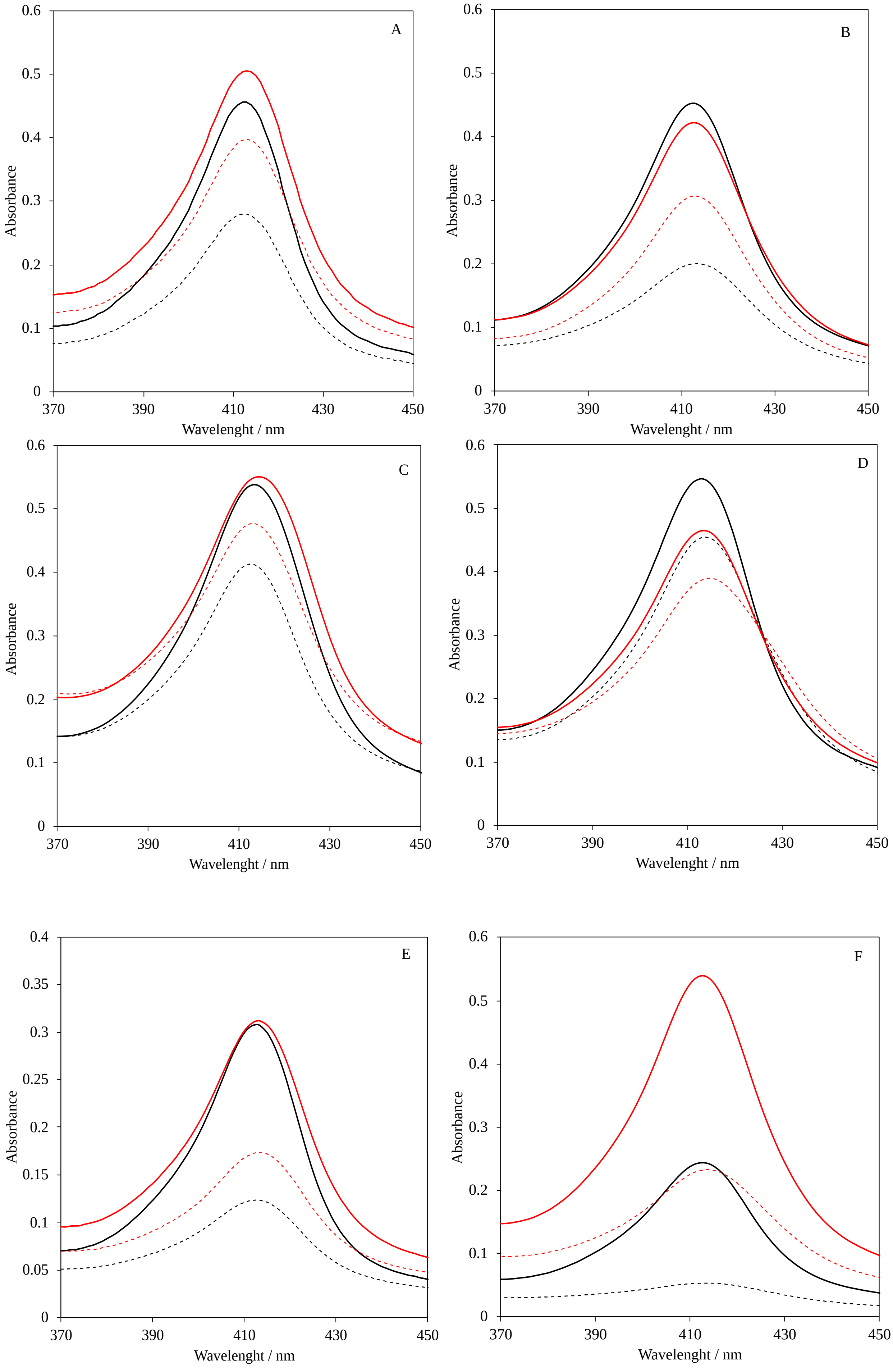
A
B
C
D
E
F
Absorbance
Wavelenght / nm
0
0.05
0.1
0.15
0.2
0.25
0.3
0.35
0.4
0.5
0.6
370
390
410
430
450

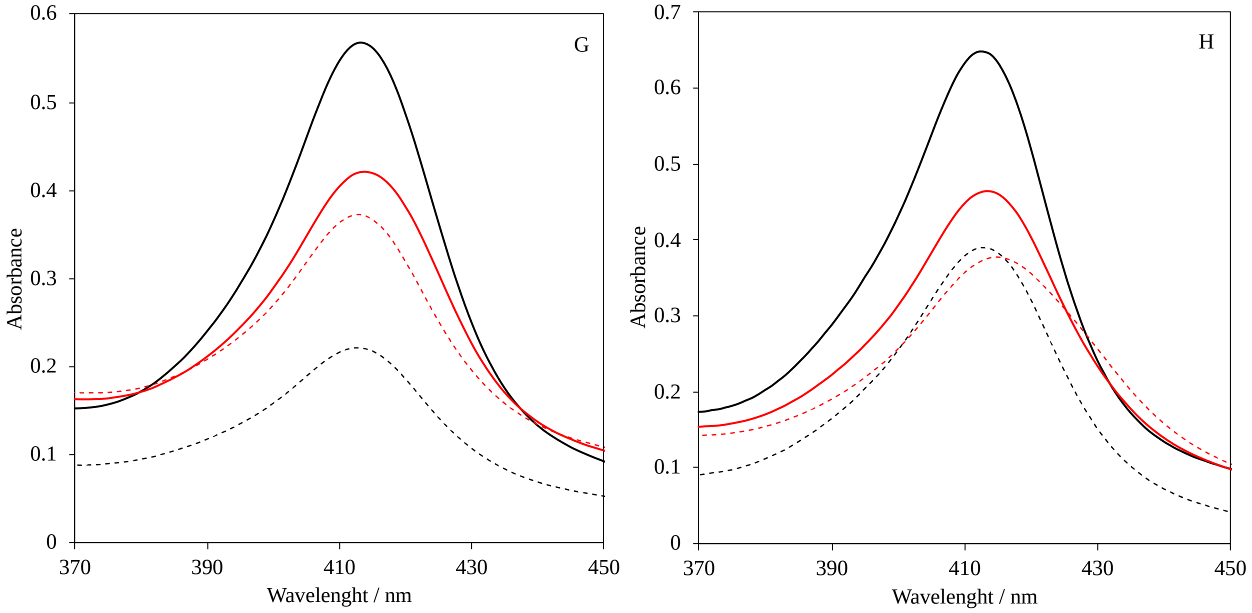

Fig. SI 2: Soret bands for spectra of hNgb and variants recorded over time after addition of H_2O_2 50 μM (A to D) and 200 μM (E to H). Continuous and dashed lines are referred to 0 hrs and 6 hrs after the injection of H_2O_2, respectively. Spectra A and E are for hNgb wt, B and F for the Y44A mutant, C and G for the Y44F mutant, and D and H for the K67A mutant with (black lines) and without (C46AC55A mutations, red lines) disulfide bridge.

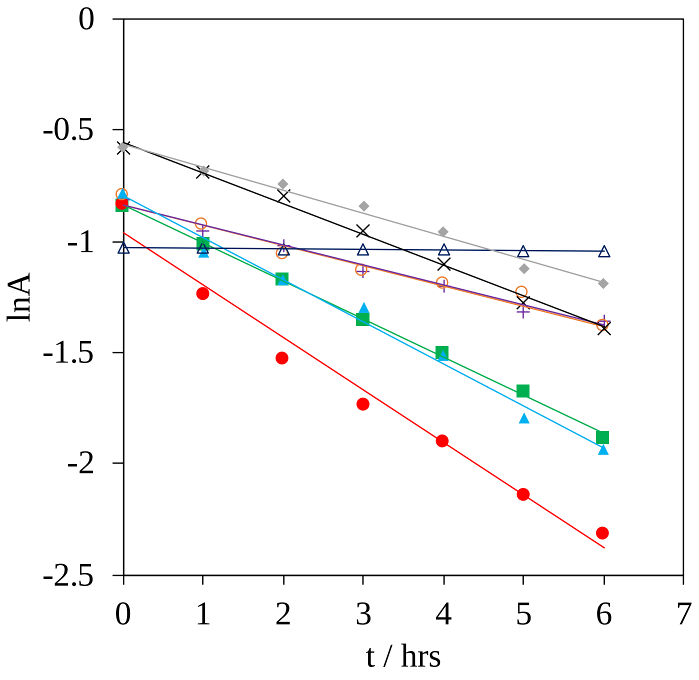

Fig. SI 3: Plot of the absorbance of the Soret band at 413 nm vs. time in the presence of 50 μM H_2O_2 (■) wt, (●) Y44A, (▲) Y44F, (×) K67A, (+) C46AC55A, (○) Y44A/C46A/C55A, (△) Y44F/C46A/C55A, (◆) K67A/C46A/C55A. Protein concentration: 3-5 μM, 10 mM phosphate buffer plus 0.1 M NaCl, pH 7.4. T = 298 K.

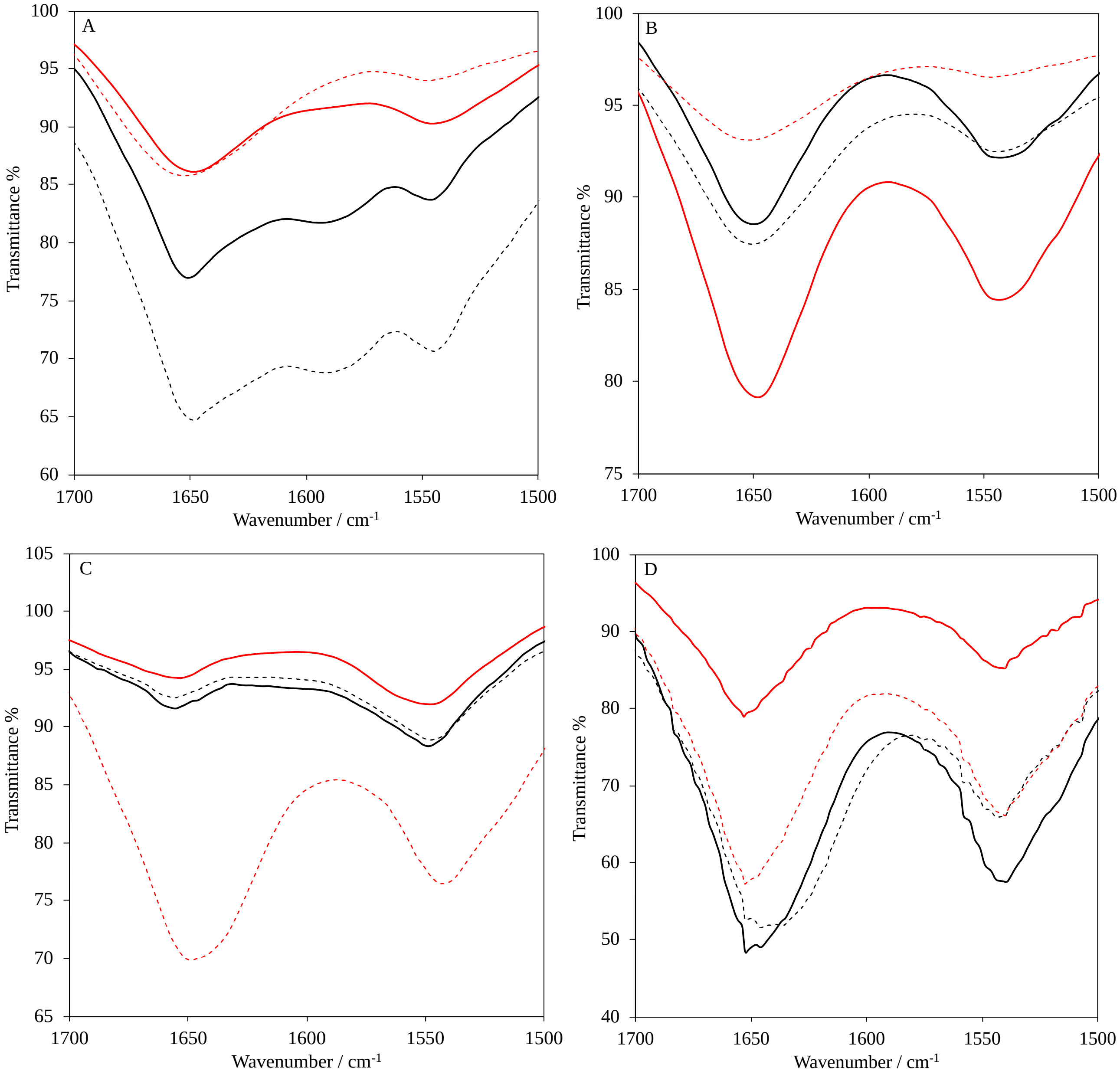

Fig. SI 4: ATR-FTIR spectra showing the Amide I and Amide II band recorded over time after addition of H_2O_2 200 μM for (A) hNgb wt, (B) Y44A, (C) Y44F, and (D) K67A mutant with (black lines) and without (C46AC55A mutations, red lines) disulfide bridge. Continuous and dashed lines are referred to 0 hrs and 24 hrs after injection of H_2O_2, respectively. Protein solutions (concentration 3 μM, pH 7.4) were subjected to Ar drying before measurement, T = 298 K.

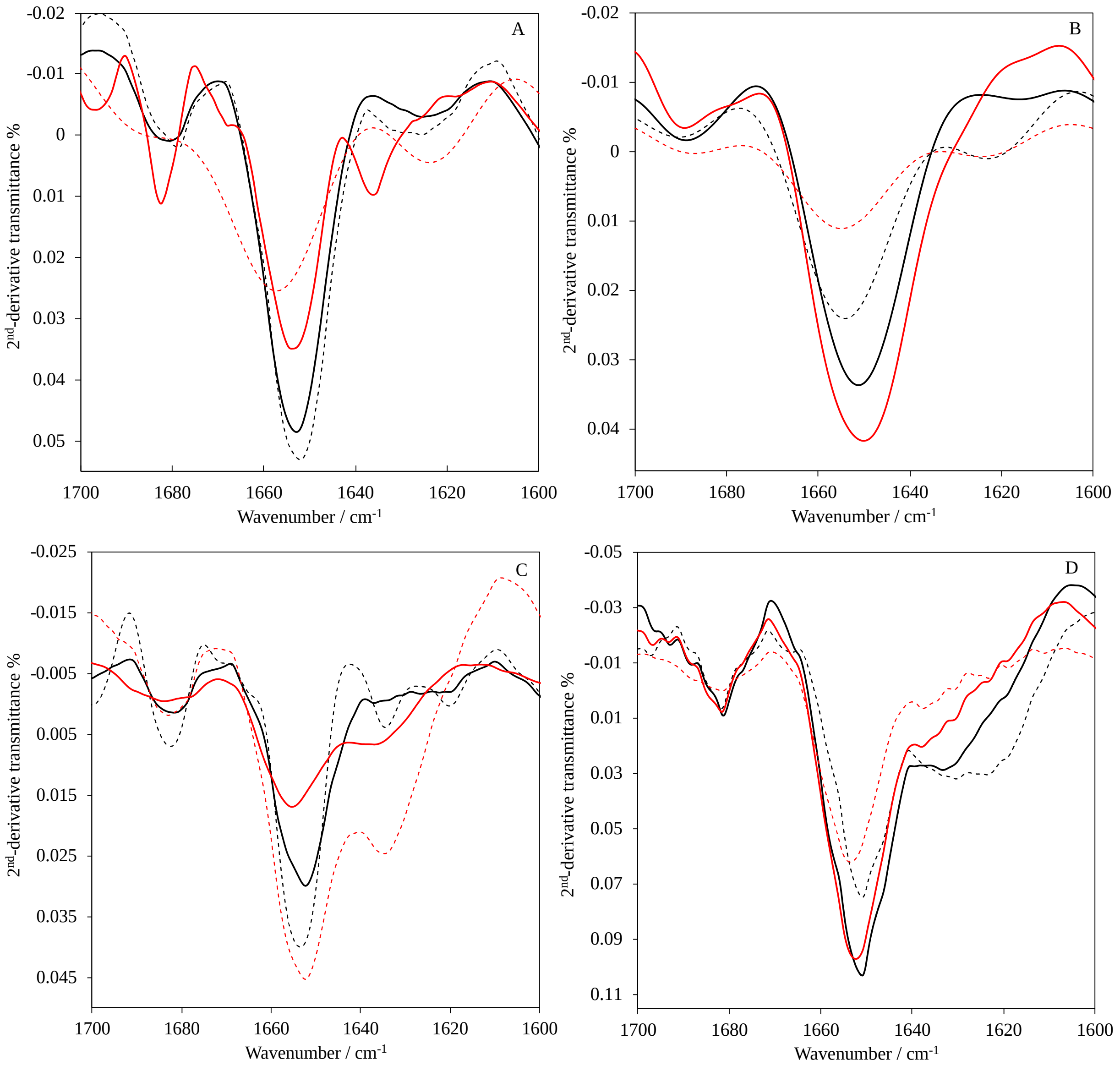

Fig. SI 5: 2nd-derivative ATR-FTIR spectra of the Amide I band recorded over time after addition of H_2O_2 200 µM for (A) hNgb wt, (B) Y44A, (C) Y44F, and (D) K67A mutant with (black lines) and without (C46AC55A mutations, red lines) disulfide bridge. Continuous and dashed lines are referred to 0 hrs and 24 hrs after injection of H_2O_2, respectively. Protein solutions (concentration 3 µM, pH 7.4) were subjected to Ar drying before measurement, T = 298 K.

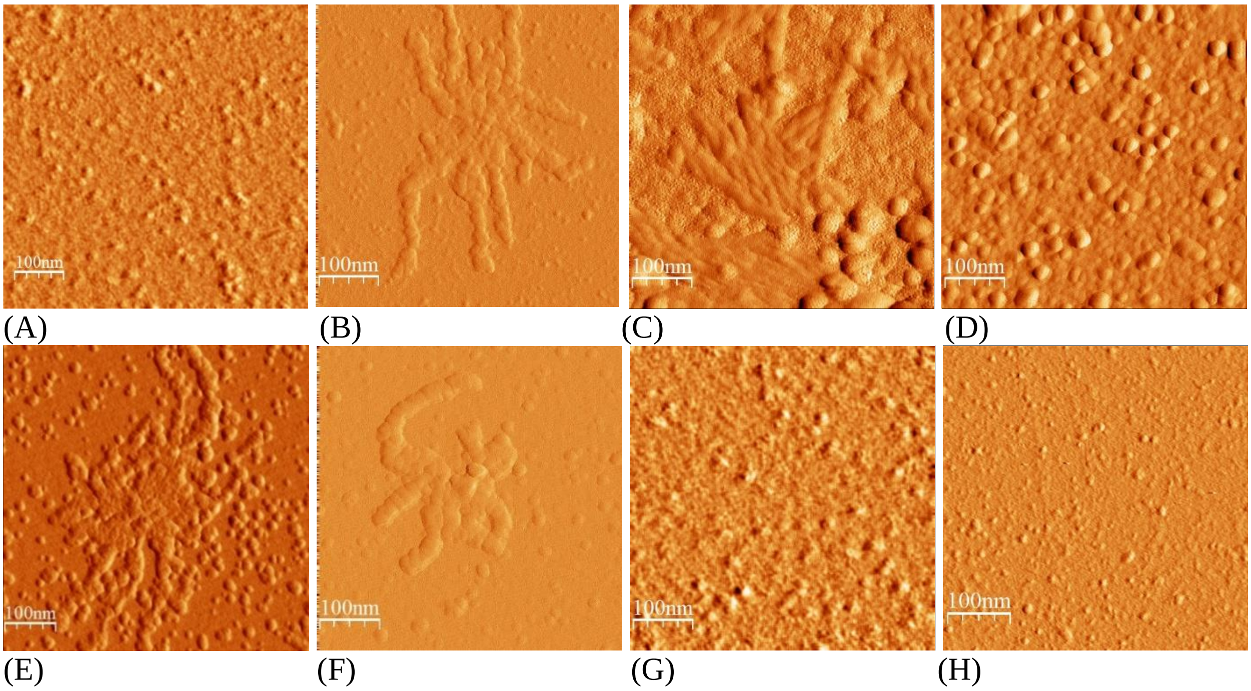

Fig. SI 6: Morphological AFM images of protein aggregates formed by hNgb and variants subjected to incubation with H_2O_2: (A) wt, (B) C46AC55A, (C) Y44A, (D) Y44AC46AC55A, (E) Y44F, (F) Y44FC46AC55A, (G) K67A, (H) K67AC46AC55A. Protein samples (5 μM in 10 mM phosphate buffer plus 0.1 M NaCl at pH 7.4 and 200 μM H_2O_2) were aged at 20 °C in the dark for 24h.

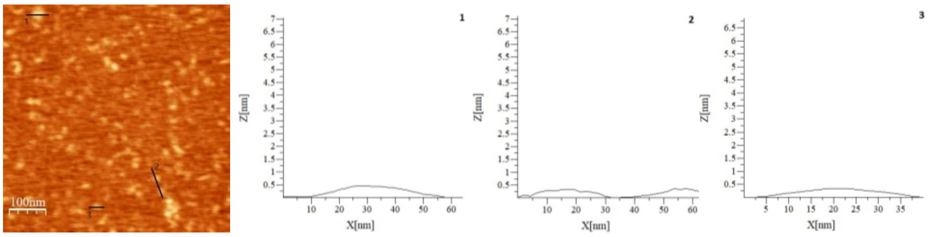
100nm
1
2
3
Z[nm]
X[nm]
(A)

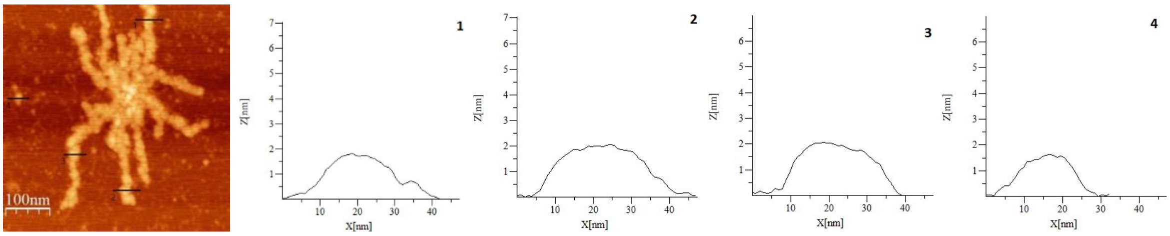
100nm
1
2
3
4
Z[nm]
X[nm]
(B)

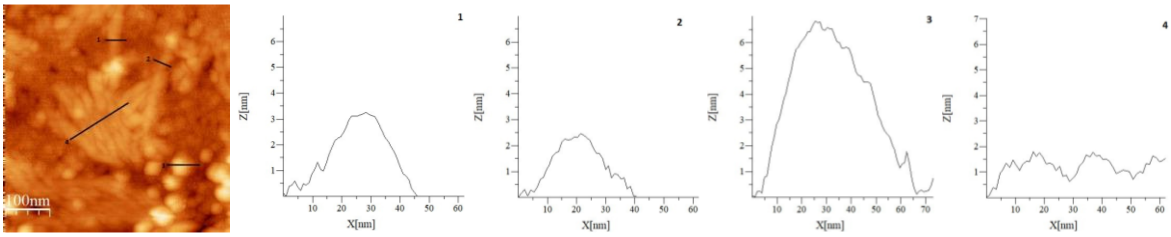
100nm
1
2
3
4
Z[nm]
X[nm]
(C)

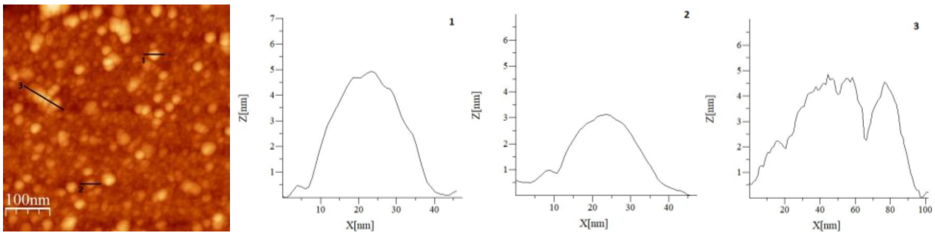
100nm
1
2
3
Z[nm]
X[nm]
(D)

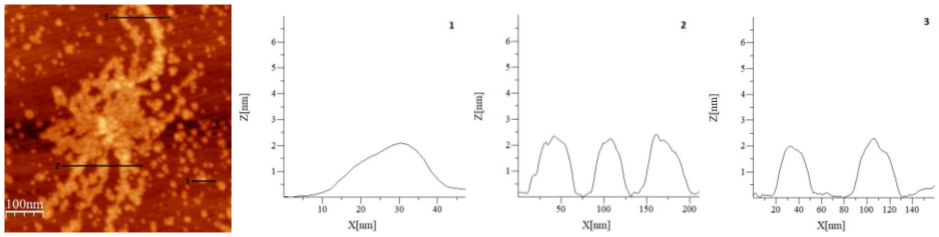

(E)

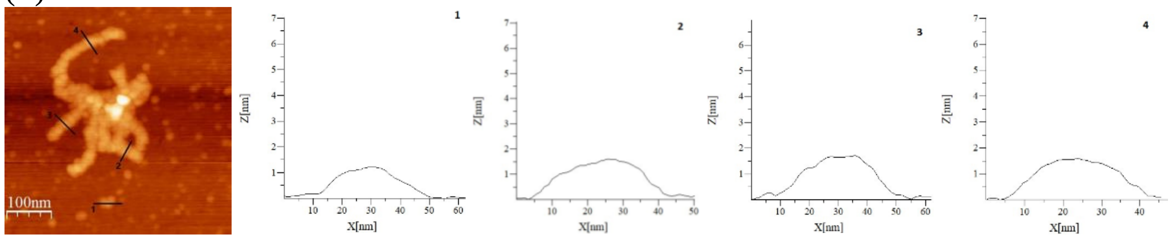

(F)

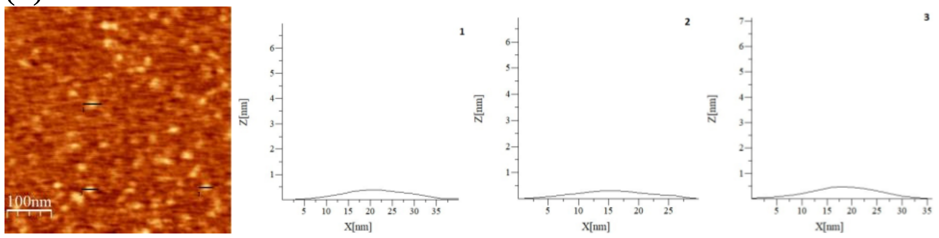

(G)

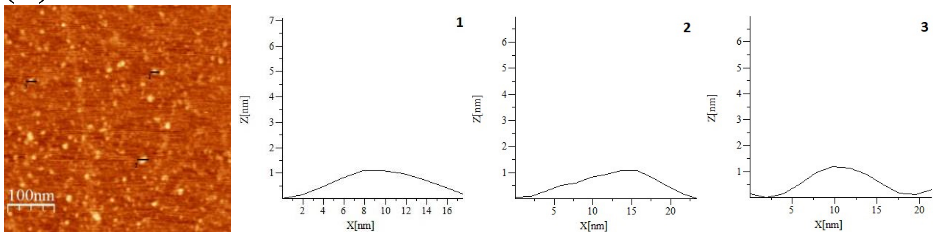

(H)

Fig. SI 7: Topographic AFM images of protein aggregates formed by hNgb (a) wt, (b) C46AC55A, (c) Y44A, (d) Y44AC46AC55A, (e) Y44F, (f) Y44AC46AC55A, (g) K67A, (h) K67AC46AC55A subjected to incubation with H_2O_2 and section height of selected aggregates. The protein samples (5 μM in 10 mM phosphate buffer plus 0.1 M NaCl at pH 7.4 and 200 μM H_2O_2) were aged at 20 °C in the dark for 24h.

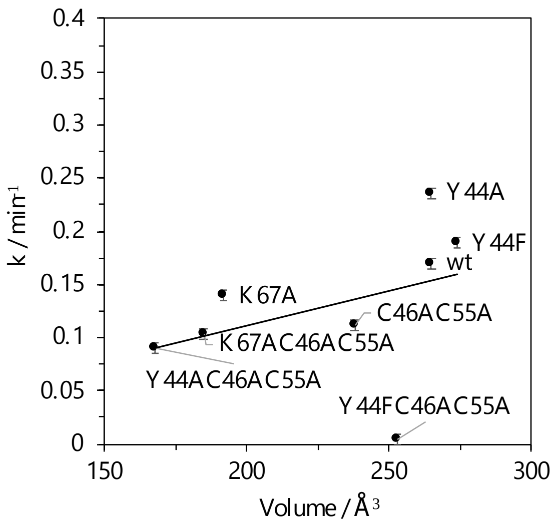

Fig. SI 8: Plot of k_{200} vs. the volume of the crevice where heme is placed (V_{hc}). V_{hc} is calculated starting from the PDB structure of hNgb wt. The cavity volume calculation does not take the heme into account.

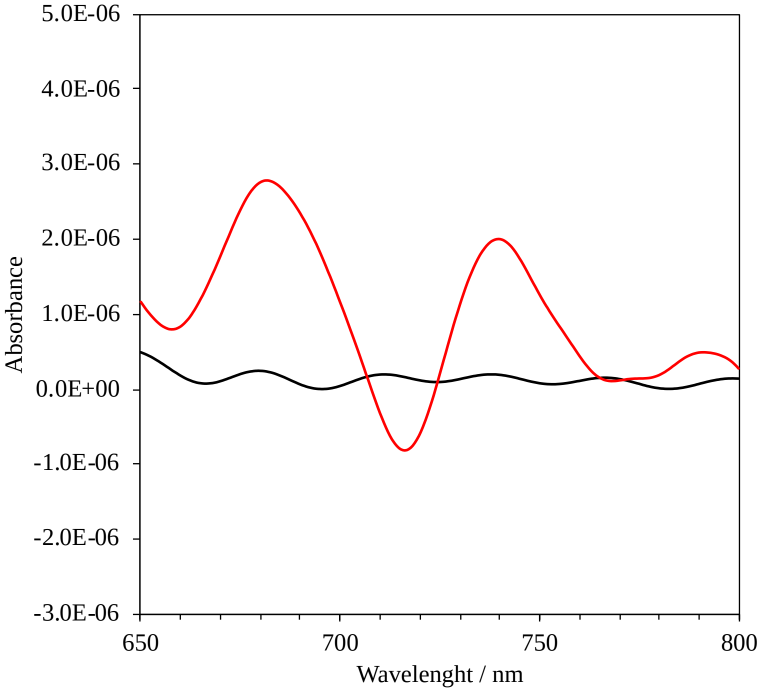

Fig. SI 9: 2nd derivative electronic absorption spectra of wt hNgb interacting with H_2O_2 at t = 0 (black) and after 6 hour incubation (red). Protein concentration: 3 μM, 10 mM phosphate buffer plus 0.1 M NaCl, pH 7.4. T = 298 K.
